# Dose Response of Acute ATP Supplementation on Strength Training Performance

**DOI:** 10.3389/fspor.2021.780459

**Published:** 2021-12-08

**Authors:** Helton Pereira dos Santos Nunes de Moura, Ralf Jäger, Martin Purpura, John A. Rathmacher, John C. Fuller, Fabrício E. Rossi

**Affiliations:** ^1^Immunometabolism of Skeletal Muscle and Exercise Research Group, Department of Physical Education and Postgraduate Program in Science and Health, Federal University of Piauí (UFPI), Teresina, Brazil; ^2^Increnovo LLC, Milwaukee, WI, United States; ^3^MTI BioTech, Inc., Ames, IA, United States; ^4^Department of Animal Science, Iowa State University, Ames, IA, United States; ^5^TSI USA LLC, Missoula, MT, United States

**Keywords:** athletic performance, adenosine triphosphate, resistance exercise, perceived exertion, pre-workout nutrition

## Abstract

**Background:** Chronic oral ATP supplementation benefits cardiovascular health, muscular performance, body composition, and recovery while attenuating muscle breakdown and fatigue. A single 400 mg dose of oral ATP supplementation improved lower body resistance training performance and energy expenditure in recreational resistance trained males, however, the minimal effective dose is currently unknown.

**Materials and Methods:** Twenty recreationally trained men (age 28.6 ± 1.0 years, body mass 81.2 ± 2.0 kg, height 175.2 ± 1.4 cm, 1RM 141.5 ± 5.0 kg) consumed a single dose of either 400 mg, 200 mg, or 100 mg ATP (PEAK ATP^®^, TSI USA LLC, Missoula, MT, USA) or a placebo in a randomized, placebo-controlled crossover design, separated by a one week wash out between treatments. After warm-up, participants performed 4 sets of half-squats using free-weights until movement failure separated by 2 mins of rest between sets.

**Results:** In comparison to placebo, 400 mg ATP significantly increased the number of set 1 repetitions (+13%, *p* = 0.04), and numerically increased total repetitions (+7%, *p* = 0.19) and total weight lifted (+6%, *p* = 0.22). 200 mg ATP numerically increased set 1 repetitions (+4% *p* = 0.47), while 100 mg ATP showed no improvements over placebo. 100 mg ATP (−4%, *p* < 0.05) and 400 mg ATP (−4%, *p* = 0.11) decreased the perceived rate of exertion compared to placebo.

**Conclusions:** In this study, the effective minimal dose of acute oral ATP supplementation during resistance exercise to increase performance was determined to be 400 mg, while as little as 100 mg showed improvements in perceived exertion.

## Introduction

All living cells use adenosine triphosphate (ATP) as a source of energy. This universal energy currency is driving the biological reactions that allow cells to function and life to exist. ATP plays a crucial role in muscle health (Jäger et al., 2021), and supplementation with ATP may improve athletic performance through increasing blood flow to the exercising muscle (Jäger et al., 2014), providing needed nutrients, and reducing fatigue.

Orally administered ATP is rapidly metabolized and acute supplementation with enteric-coated ATP is not bioavailable (Arts et al., 2012). However, oral supplementation with ATP results in increased capacity to synthesize ATP in red blood cell pools (Jäger et al., 2014). During times of increased energy expenditure, ATP supplementation has been shown to prevent declines in ATP levels, providing additional energy and delaying fatigue (Purpura et al., 2017).

A recent review of studies where oral ATP was supplemented has shown multiple benefits like cardiovascular health, muscular performance, body composition, and recovery while attenuating muscle breakdown and fatigue (Jäger et al., 2021). In a study where 12 weeks of resistance training exercise was combined with 400 mg of disodium ATP supplementation in healthy, resistance-trained males, ATP supplementation resulted in significantly greater increases in lean mass, muscle thickness, maximal strength, and vertical jump power in comparison to exercise alone (Wilson et al., 2013). In addition, ATP supplementation was able to attenuate losses of strength and power and reduced muscle damage during a two-week overreaching period (Wilson et al., 2013). In another study, 15 days of 400 mg disodium ATP supplementation resulted in better maintained performance during the latter bouts of repeated bouts of maximal cycling performance compared to placebo supplementation (Purpura et al., 2017).

While certain ergogenic sports supplements, like creatine (Kreider et al., 2017) or beta-alanine (Trexler et al., 2015), need to be consumed daily for several weeks to increase athletic performance, a recent study has shown that ATP works acutely (Freitas et al., 2019). Thirty minutes after ingestion, participants completed a series of half-squat repetitions at 80% of their 1RM, and in comparison, to placebo supplementation, the total weight lifted was significantly increased in the ATP group compared to the placebo group. Acute ATP supplementation has also been shown to improve blood pressure in hypertensive women after just a single dose of 400 mg. The test subjects performed 30 mins of aerobic exercise at 70–75% of their individual maximum heart rate. ATP induced faster recovery of heart rate variability and reduced systolic blood pressure after aerobic exercise (Freitas et al., 2018).

Most recent studies have supplemented disodium ATP at 400 mg while earlier studies used lessor amount. However, the optimal and minimally effective dose of ATP to increase exercise performance is currently unknown. Therefore, the current study was designed to investigate asministration of acute dosages of either 100, 200, or 400 mg of ATP disodium supplementation on resistance exercise, which was conducetd until movement failure, and when compared to placebo, determine a minimally effective dosage.

## Materials and Methods

### Experimental Design

**Figure 1:**
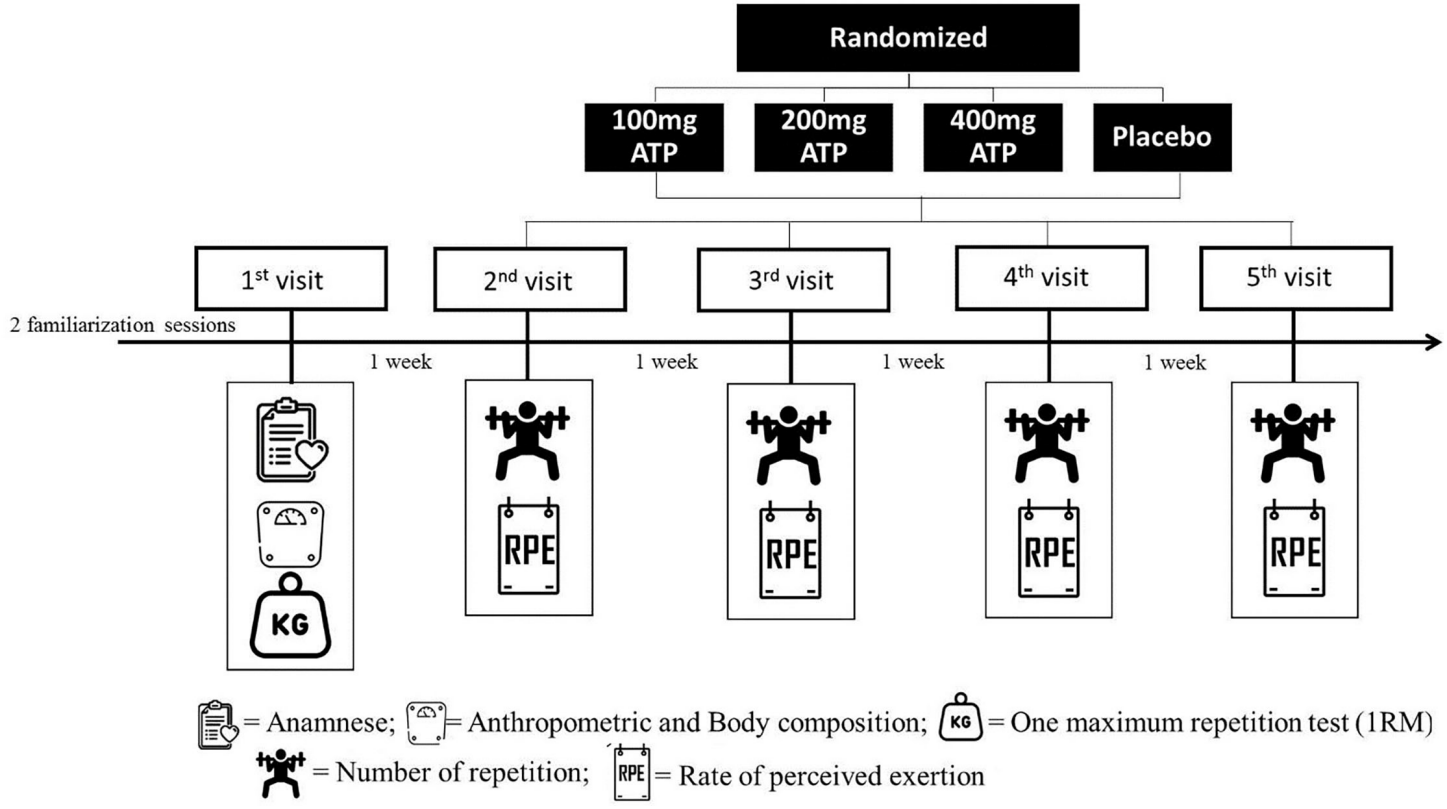
Study design.

The study was conducted using a randomized, double-blind, placebo-controlled crossover study design. Subjects completed 5 experimental trials at the laboratory, each separated by a one-week washout period. All trials were performed at the same time (morning) to ensure chronobiological control. A schematic illustration of the experimental design is shown in Figure 1. Anthropometric measurements and 1RM test for half-squats was determined during the first visit. Height was measured on a fixed stadiometer (Sanny, American Medical, São Bernardo do Campo, São Paulo, Brazil), with an accuracy of 0.1 cm and a length of 2.20 m. Body mass was measured using an electronic scale (Filizola PL 50, Filizola Ltd., Brazil), with a precision of 0.1 kg. The subjects were asked to avoid alcoholic beverages for at least 12 hours before the test and came to the lab between 8 and 9 am after an overnight fast. The participants were instructed to remove metal items from their body and were positioned in a supine position with their arms forming an angle of approximately 30° and their legs forming an angle of 45°. Subjects remained still throughout the examination and were wearing light clothing (Mialich et al., 2014). The fat-free mass (FFM) and fat mass (FM) in kilograms, and percentage of fat mass (FM%) were assessed using spectral bioelectrical impedance analysis and accompanying software (Biodynamics model 310e, Biodynamics Corporation, Shoreline, WA, United States). On the following 4 visits, each subject consumed randomly either 100, 200, or 400 mg of ATP (PEAK ATP^®^, TSI USA LLC, Missoula, MT, USA) or a matching placebo (maltodextrin). The three Peak ATP treatments and the placebo treatment were assigned a random number using random numbers generated in Excel. All treatments were packaged into foil sachets and labeled with the assigned random number and the lot number by the TSI Group LTD (Shanghai, China). The placebo powder was identical to the ATP treatments in weight, texture and color. The supplements in each group were matched for total quantity of capsules, e.g., the 200 mg ATP group received two 100 mg ATP capsules and two 100 mg placebo capsules. The supplement or placebo was ingested 30 mins prior to the resistance exercise tests. The order of administration was randomized using an online randomization software program (www.randomizer.org). Participants completed 4 sets of half-squats using free-weights until momentary muscular failure with a load corresponding to 80% of the 1RM and 2 mins of rest between sets. Subjects were required to reach parallel squats, from complete knee extension to approximately 90° knee flexion and then immediately returned to the extended knee for the attempt to be considered successful as determined by the research team (Silva et al., 2017) and the participants performed resistance exercise sets to momentary muscular failure (i.e., the maximum number of possible repetitions in a given set) (Fisher and Steele, 2013). The total number of repetitions performed was recorded for each set and was used to analyze performance and the rate of perceived exertion was measured at the end of each session of exercise using the OMNI Perceived Exertion Scale for Resistance (Lagally and Robertson, 2006). Adverse gastrointestinal events were monitored after each trial through questioning of the participants by the researchers: “Do you have any adverse gastrointestinal symptoms, i.e., nausea, stomach ache, intestinal cramps and or the urge to vomit?”

### Subjects

Prior to beginning the study, all participants signed an IRB-approved informed consent document (Federal University of Piauí, Terezina, PI, Brazil. Protocol number: 3.169.545, approval date: 02/26/2019) and completed a healthy history questionnaire to determine study eligibility. This study protocol and design was retrospectively registered with the ISRCTN registry on July 8, 2021 as study record ISRCTN96148141. Food questionnaires were distributed to all participants to record their food and fluid intake at pre-exercise meal (breakfast) 90 min prior to each trial. Participants were instructed to replicate the first trial's dietary intake for the subsequent trial. All food intakes were analyzed for total kilocalorie and macronutrient intakes using MyFitnessPal.com (http://www.myfitnesspal.com) and shown in the Table 1.

**Table 1:** Dietary intake and macronutrient distribution pre-exercise.

**CHO (g)**	**46.2 ± 18.4**
**PRO (g)**	**26.8 ± 13.6**
**FAT (g)**	**20.6 ± 8.2**
**Total Intake (kcal)**	**492.4 ± 129.5**

*CHO, carbohydrate; PRO, protein; FAT, lipids*.

**Figure 2:**
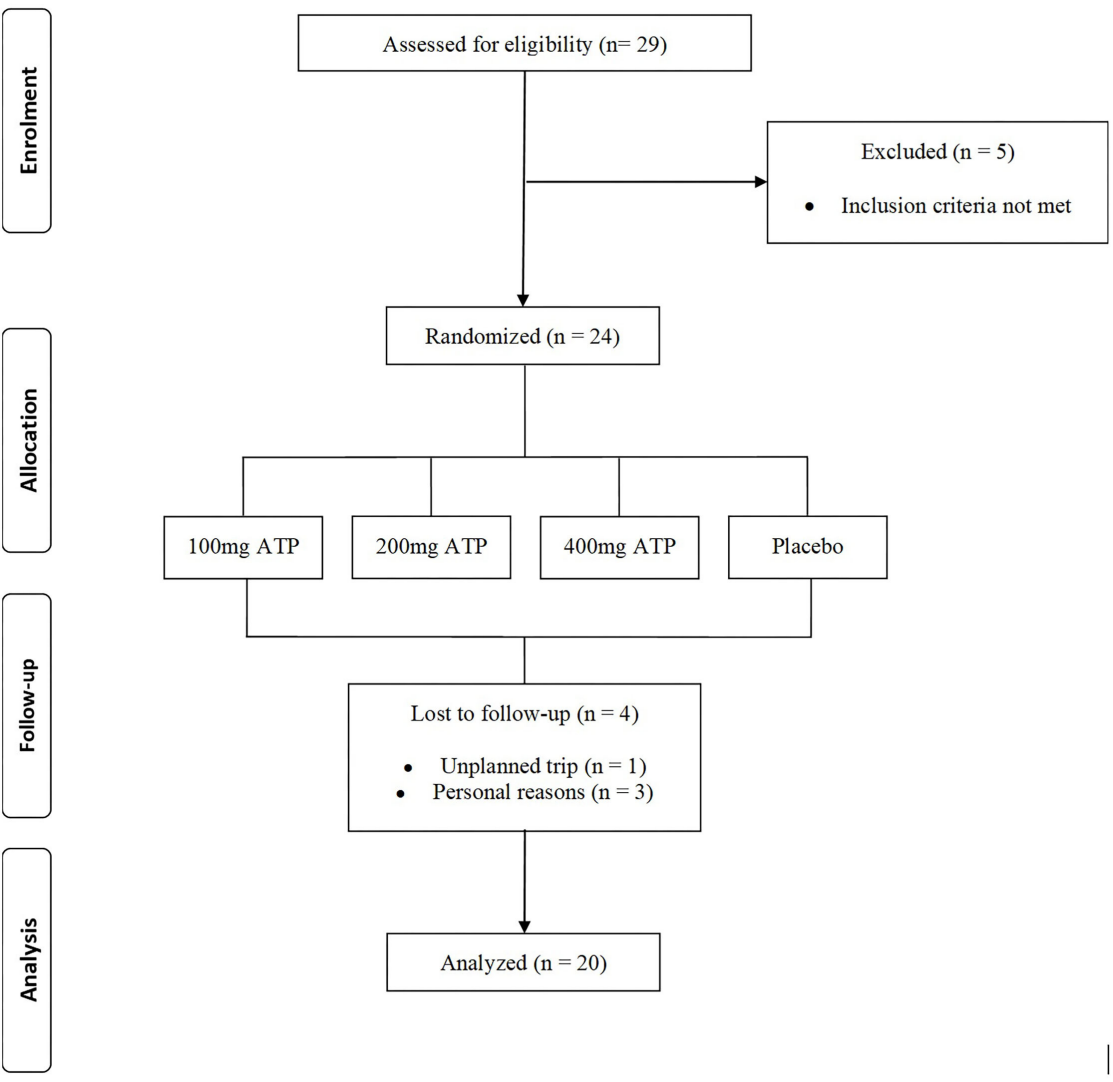
CONSORT Chart.

A power analysis (G-Power, v3.0, Universität Kiel, Germany) was completed based on preliminary repetition data from this study. The power analysis was based on a t-test for independent means with an effect size of 0.61 with an α error probability of 0.05, and power of 0.8, it was estimated that 18 participants would be needed in the cross-over design per treatment. Out of a total of 29 men who participated in the first screening, 5 failed the screening (due to either taking ergogenic supplements, or not matching the required relative 1RM of 1.5 to 2.0 kg/body weight, or because they were not available to visit the lab 4 more times in the requested timeframe) and 24 healthy subjects who met all the inclusion criteria were enrolled into the study. During the intervention, four participants dropped out due to an unplanned trip during the study (*n* = 1), or personal reasons (*n* = 3) after completing either the first or second visit (see Figure 2). 20 recreationally resistance-trained males successfully completed all study visits (see Table 2 for full participant characteristics).

**Table 2:** Participant characteristics (means ± standard error of the mean).

**Age (years)**	**28.6 ± 1.0**
**Weight (kg)**	**81.2 ± 2.0**
**Height (cm)**	**175.2 ± 1.4**
**Fat Mass (kg)**	**14.2 ± 1.0**
**Fat Free Mass (kg)**	**67.0 ± 1.4**
**Fat (%)**	**17.2 ± 0.9**
**1RM (kg)**	**141.5 ± 5.0**
**Relative 1RM (kg)**	**1.73 ± 0.04**

Inclusion criteria for participation in the study included age (18–35 years of age), gender (male), healthy and free of disease (as reported by the health screening questionnaire), and physically active with at least 1 year of resistance training experience (3 days per week for at least 60 mins per day), and a relative 1RM of 1.5 to 2.0 kg/body weight. Any individual diagnosed with or being treated for cardiac, respiratory, circulatory, musculoskeletal, metabolic, obesity (defined as body mass index >30 kg/m^2^ and body fat >30%), immune, autoimmune, psychiatric, hematological, neurological, or endocrinological disorder or disease, or taking dietary supplements 6 months prior to enrolling into the study that could influence the outcome measures of this study were excluded from participating. Subjects were instructed not to change their regular diet and exercise patterns, not to consume any ergogenic substances during the duration of the study and were not allowed to consume caffeine for 12 h before each experimental test.

### Resistance Exercise Protocol

Resistance exercise was performed identical to previously described (Trexler et al., 2015). The reliability of the test method was determined in a pilot study (*n* = 6). The test-retest Intraclass Correlation Coefficient (ICC) was determined to be Set-1 (0.97), Set-2 (0.92), Set-3 (0.95) and Set-4 (0.91). Before their first trial, subjects completed two familiarization sessions to become acquainted with the 1RM test procedures and training equipment. Before 1RM testing, subjects completed a warm-up protocol, which consisted of 5 mins of walking and subsequent 1 set of 10 repetitions at approximately 50% of the 1RM. The load was increased gradually (10–15%) during the test until the participants were no longer able to perform the entire movement, and 3–5 attempts. Before the experimental trials subjects performed a warmup with walking for 5 mins on a treadmill and a subsequent 1 set of 15 repetitions at 30% of 1RM. After 3 mins of recovery, each participant completed 4 sets until movement failure at 80% of 1RM with normal speed (1-s eccentric and 1-s concentric) and 2 mins of rest intervals between sets. Two fitness professionals supervised all testing sessions. During the exercise sessions, subjects were verbally encouraged to perform all sets to exhaustion. For better control of the strength test procedures and resistance exercise protocol, a wooden seat with adjustable heights was placed behind the participant to keep the bar displacement and knee angle constant on each repetition.

### Statistical Analyses

Statistical analyses were performed for the number repetitions in Set-1 Set-2, Set-3, and Set-4, total repetitions, total weight lifted, and perceived rate of exertion. Data sets for each variable were analyzed for homogeneity of variance using Levene's test followed by the Shapiro–Wilk test for normality. No transformations were of the data sets were performed nor was there missing data. All data were analyzed using the General Linear Model ANOVA of SAS (Version 9.4, SAS Institute Inc., Cary, NC), using a cross-over statistical model that included subject, order, and treatment main effects for balanced data. *Post-hoc t*-tests were performed on the Least Squares-Means where significant model effects or interactions were observed. Statistical significance was determined for *p* < 0.05. All data are expressed as Least Squares-Means ± standard error.

## Results

**Figure 3:**
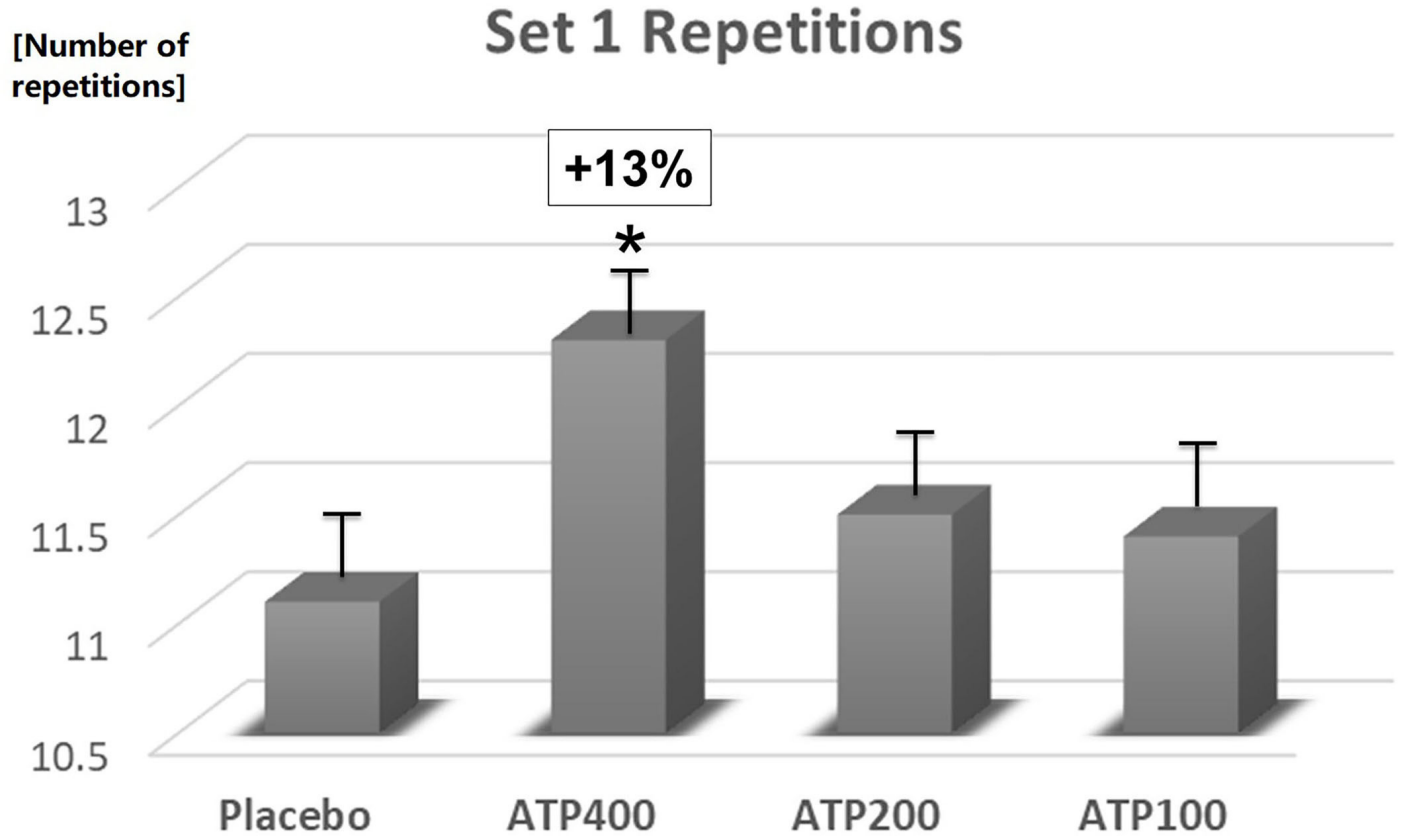
400 mg ATP significantly (*p < 0.04) increased the number of repetitions in set 1. Data are expressed as Least Squares-Means ± standard error.

**Figure 4:**
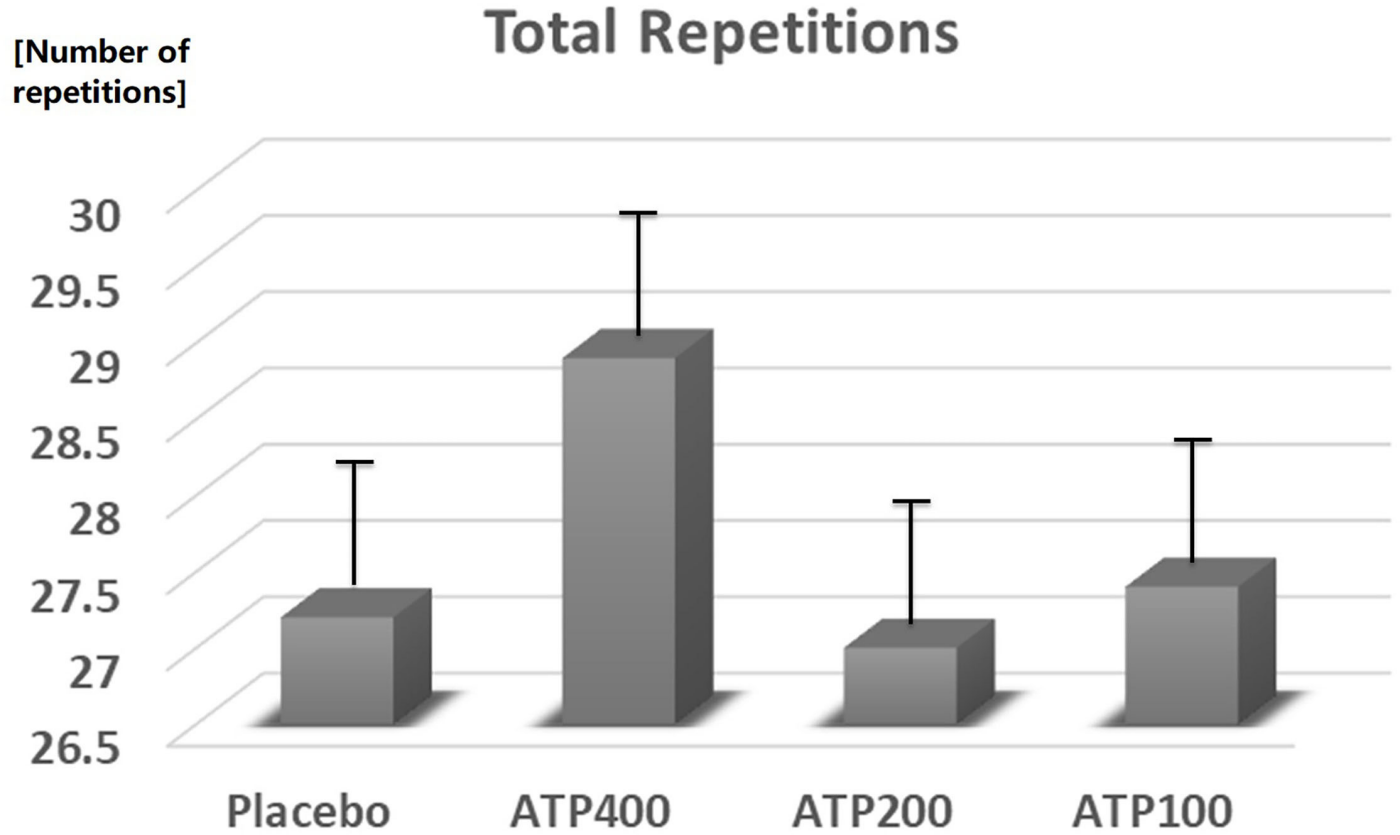
ATP supplementation did not statistically significant increase the total number of repetitions. Data are expressed as Least Squares-Means ± standard error.

**Figure 5:**
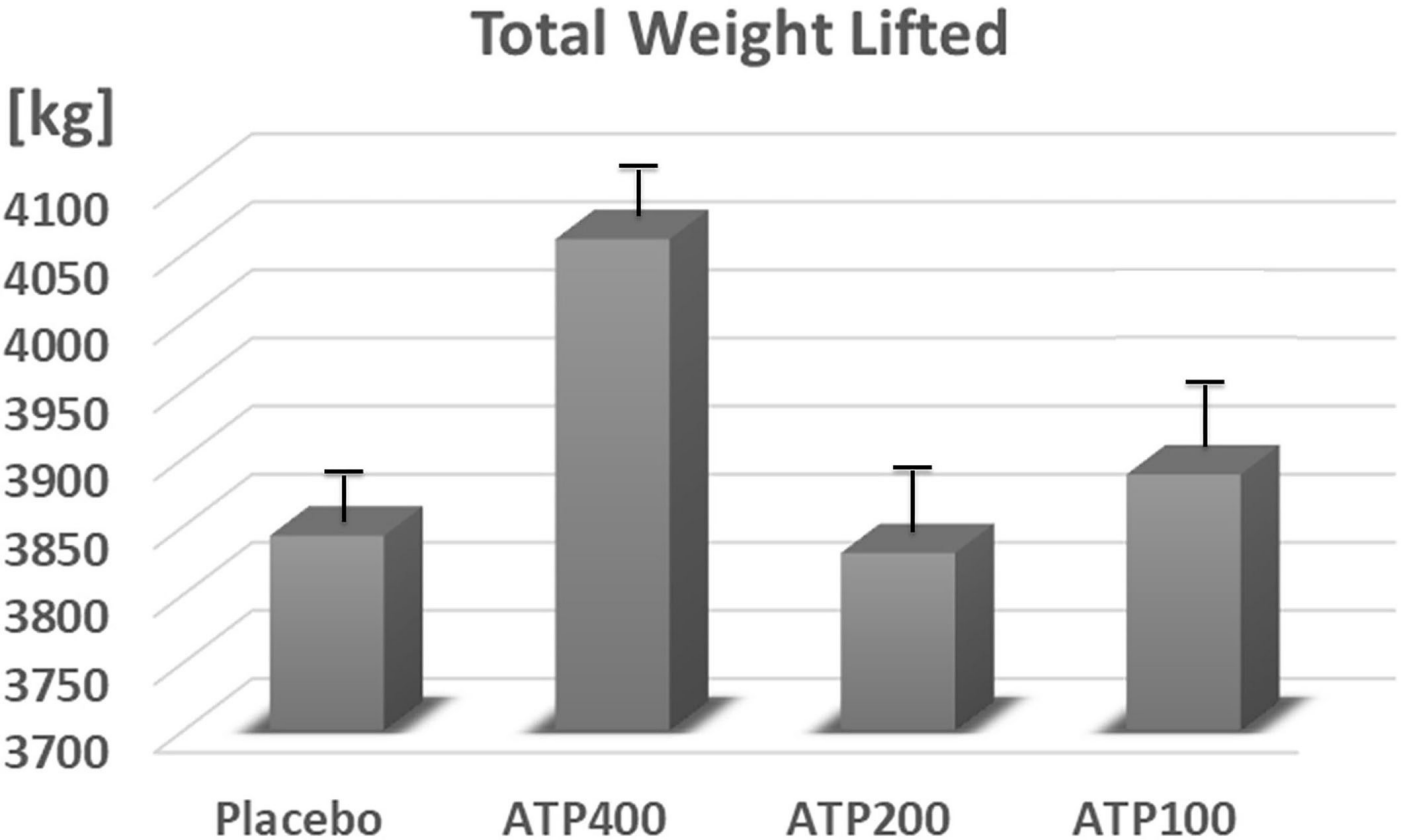
ATP supplementation did not statistically significant increase the total amount of weight lifted. Data are expressed as Least Squares-Means ± standard error.

In comparison to placebo, 400 mg ATP significantly increased the number of set 1 repetitions (11.1 ± 0.4 vs. 12.3 ± 0.4, +13%, *p* = 0.04, see Figure 3), and numerically increased total repetitions (27.2 ± 0.9 vs. 28.9 ± 0.9, +6.3%, *p* = 0.19, see Figure 4) and total weight lifted (3,842.8 ± 121.6 kg vs. 4,060.5 ± 121.6, +6%, *p* = 0.22, see Figure 5). Two hundred mg ATP numerically increased set 1 repetitions (11.1 ± 0.4 vs. 11.5 ± 0.4, +4%, *p* = 0.47), while 100 mg ATP increased set 1 repetitions by 3% over placebo (11.1 ± 0.4 vs. 11.4 ± 0.4, *p* = 0.63, see Figure 3). Two hundred mg and 100 mg had no effect on total repetitions and total weight lifted (see Figures 4, 5).

**Figure 6:**
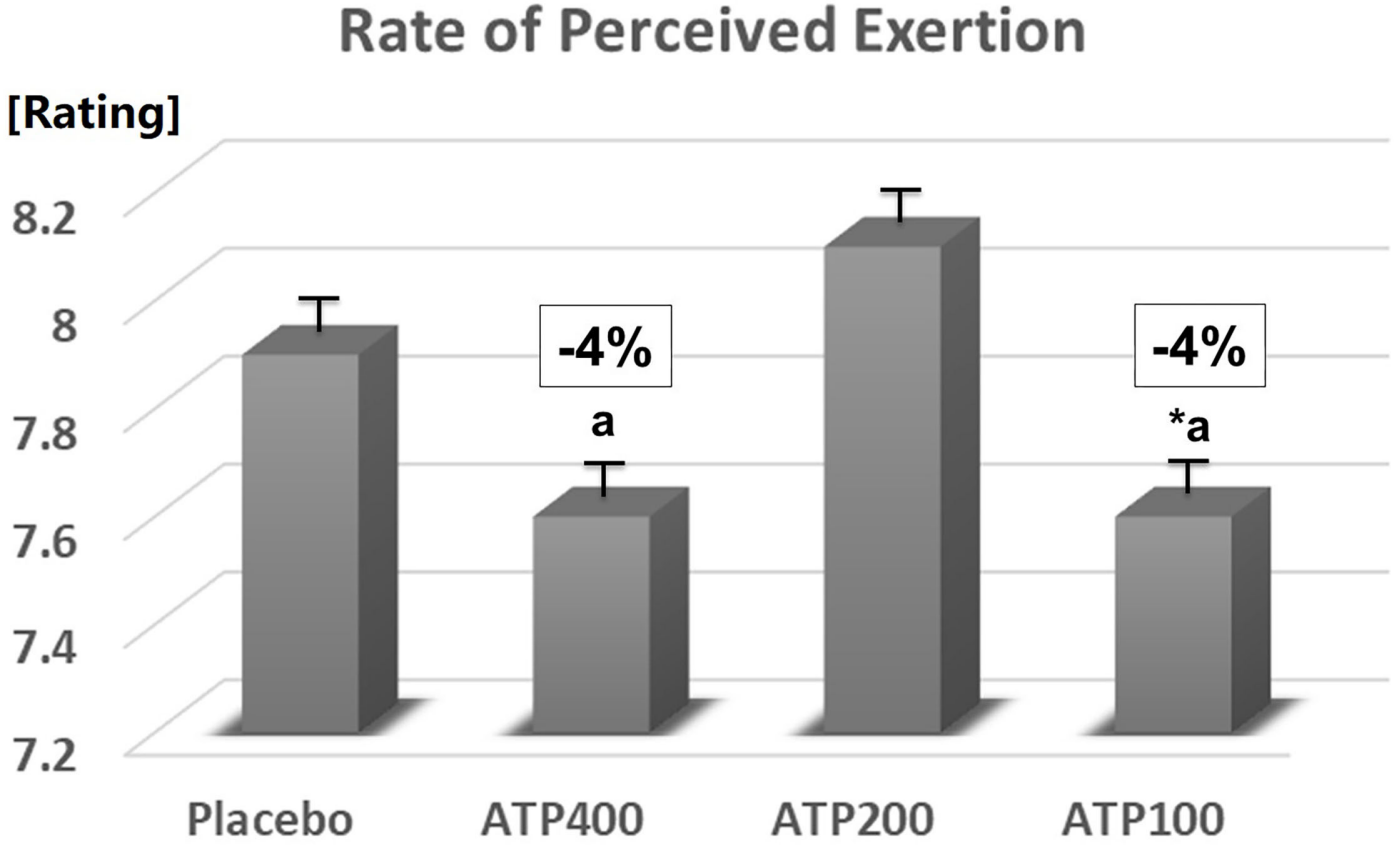
100 mg ATP decreased the rate of perceived exertion (*p < 0.05 significantly different from placebo). Data are expressed as Least Squares-Means ± standard error.

Supplementation at 100 mg ATP (−4%, *p* < 0.05) and 400 mg ATP (−4%, *p* = 0.11) decreased the perceived rate of exertion compared to placebo and compared to 200 mg ATP (*p* < 0.05) (see Figure 6).

None of the participants reported any adverse gastrointestinal effects from ingesting either the ATP dosage or placebo.

## Discussion

The prevention of declines in ATP pools during times of increased energy needs through oral supplementation of ATP allows athletes to maintain performance through longer periods of exertion and consequently delay the onset of fatigue (Purpura et al., 2017). Chronic ATP supplementation in combination with resistance exercise further increases the beneficial effects of exercise on strength, power, and body composition (Wilson et al., 2013). Our dose response study confirmed previous findings, that a single-dose of 400 mg ATP 30 mins prior to exercise can increase performance during lower-body resistance exercise in recreationally trained men (Freitas et al., 2019). Lower doses failed to show significant performance benefits, but showed a beneficial effect on the rate of exertion with 100 mg ATP supplementation compared to placebo.

Previous studies investigating the effects of chronic ATP supplementation on athletic performance differ in the training status of the participants, the timing of the supplementation, and the type of exercise (Jordan et al., 2004; Rathmacher et al., 2012; Wilson et al., 2013; Purpura et al., 2017). Rathmacher et al. (2012) reported that 14 days of 400 mg/day ATP supplementation led to improved ability to maintain a higher force output during the final 10 repetitions of an exhaustive 50-repetition exercise bout. In contrast to our study, participants did not consume ATP on the day of the tests, and they did not observe any improvements in performance in early sets (Rathmacher et al., 2012). Low-dose ATP supplementation (150 or 225 mg enteric-coated ATP per day) for 15 days in untrained individuals with the final dose being taken 3 hours prior to exercise increased total bench press lifting volume as well as within-group set 1 repetitions to failure (Jordan et al., 2004). In another study, 400 mg of ATP taken for 15-days, with the final dose taken 30-mins prior to exercise has been shown to increase early set performance in a repeated sprint cycling exercise, as well as reducing drops in performance in later bouts (Purpura et al., 2017).

Possible mechanisms by which single-dose ATP supplementation enhances resistance exercise performance include changes in blood flow (Jäger et al., 2014), increasing intracellular calcium influx (Sandonà et al., 2005), preventing exercise-induced declines in ATP levels (Purpura et al., 2017), and increased energy expenditure (Freitas et al., 2019). Exercise increases blood flow to the exercising muscle, providing nutrients and oxygen and removing waste products. Changes in blood flow produce “shear stress” on the endothelial cells lining blood vessel walls, causing the cells to release ATP, which activates receptors on nearby endothelial cells (Khakh and Burnstock, 2009). The cells respond by releasing nitric oxide, which makes the vessels relax (Jäger et al., 2021). Supplemental ATP has been clinically shown to further increase this natural response to exercise. Twelve weeks of 400 mg of ATP supplementation significantly increased blood flow and significantly enhanced brachial artery dilation following resistance exercise (Jäger et al., 2014). Muscle fatigue and a reduction in force production is linked to reduced calcium release by the sarcoplasmic reticulum and by binding to the P2X4 receptor, ATP increases intracellular calcium influx (Sandonà et al., 2005). Supplementation with ATP might delay the reduction of calcium release during muscle contractions, improving muscle strength production. While ATP has been shown to act through multiple mechanisms as described, in our study acute administration likely works through a more immediate response in red blood cell ATP pools and blood flow to the muscles.

Limitations of the present study include the lack of dietary control, since the habitual food intake in the last 24–48 h are a risk of bias involved in the analyzed variables, and the lack of assessments on calcium influx or blood flow, another potential mechanism by which ATP could increase lower-body resistance exercise in recreationally trained men and the lack of post-exercise heart rate measurements, as a more objective measure of rate of perceived exhaustion. Future studies could investigate if the acute physical performance benefits translate to acute cognition benefits, as ATP production and utilization play a major role in cerebral bioenergetics and brain function (Du et al., 2018), and if the benefits seen in weight trained athletes translate into endurance type exercise, or older populations. In addition, further studies could test if the observed improved perception of exertion with the low 100 mg dose used during a chronic supplementation protocol might allow athletes to train harder and more frequently, resulting in faster training adaptations and increased training benefits. More well-design studies are needed to corroborate the presented findings.

Therefore, our findings confirmed that acute supplementation with 400 mg ATP increased performance in recreationally trained males during lower body resistance exercise, however, more research is needed to investigate if the numerical changes observed in the lower dose groups might translate in meaningful long-term performance benefits, when supplemented chronically in combination with resistance training exercise.

## Conclusion

The effective dose of acute oral ATP supplementation during resistance exercise to increase performance was determined to be 400 mg.

## Data Availability Statement

The raw data supporting the conclusions of this article will be made available by the authors, without undue reservation.

## Ethics Statement

The studies involving human participants were reviewed and approved by Ethics Committee of the Federal University of Piaui. The patients/participants provided their written informed consent to participate in this study.

## Author Contributions

FR, RJ, MP, JF, and JR: conceptualization. FR and HS: investigation. JR: statistical analysis. RJ and FR: writing—original draft. All authors have read and agreed to the published version of the manuscript.

## Funding

This study was funded by TSI USA LLC, Missoula, MT, USA. The funder was not involved in the collection of data or the decision to submit it for publication.

## Conflict of Interest

RJ and MP were employed by company Increnovo LLC. JR was employed by company MTI BioTech, Inc. This study received funding from TSI USA LLC, Missoula, MT, USA. JF is employed by TSI. RJ and MP are paid consultants to TSI. JR was involved in the study design and statistical analysis of data. JF and MP were involved in the study design. RJ was involved in study design and writing of the manuscript. The remaining authors declare that the research was conducted in the absence of any commercial or financial relationships that could be construed as a potential conflict of interest.

## Publisher's Note

All claims expressed in this article are solely those of the authors and do not necessarily represent those of their affiliated organizations, or those of the publisher, the editors and the reviewers. Any product that may be evaluated in this article, or claim that may be made by its manufacturer, is not guaranteed or endorsed by the publisher.
